# The ASCENT Consortium: A New Resource to Support Palliative Care Science Across the Lifespan

**DOI:** 10.1016/j.jpainsymman.2026.02.003

**Published:** 2026-02-11

**Authors:** Jean S. Kutner, Melissa D. Aldridge, Abraham A. Brody, Chris Feudtner, Kimberly Johnson, Stacy M. Fischer, Susan Lysaght Hurley, Alexis Bakos, Elena M. Fazio, Karen A. Kehl, Sandra A. Mitchell, Elizabeth A. Necka, Brennan Parmelee Streck, Chandra Keller

**Affiliations:** University of Colorado School of Medicine, Anschutz Medical Campus, Aurora, Colorado, USA; Brookdale Department of Geriatrics and Palliative Medicine, Icahn School of Medicine at Mount Sinai, New York, New York, USA; New York University Rory Meyers College of Nursing, New York, New York, USA; Division of General Pediatrics, Children’s Hospital of Philadelphia, Philadelphia, Pennsylvania, USA; Department of Medicine, Division of Geriatrics, Duke University School of Medicine, Durham, North Carolina, USA; University of Colorado School of Medicine, Anschutz Medical Campus, Aurora, Colorado, USA; New York University Rory Meyers College of Nursing, New York, New York, USA; Care Dimensions Inc, Danvers, Massachusetts, USA; National Institute on Aging, National Institutes of Health, Bethesda, Maryland, USA; National Institute on Aging, National Institutes of Health, Bethesda, Maryland, USA; National Institute of Nursing Research, National Institutes of Health, Bethesda, Maryland, USA; National Cancer Institute (NCI), National Institutes of Health, Bethesda, Maryland, USA; National Institute of Mental Health, National Institutes of Health, Bethesda, Maryland, USA; National Cancer Institute (NCI), National Institutes of Health, Bethesda, Maryland, USA; National Institute on Aging, National Institutes of Health, Bethesda, Maryland, USA

**Keywords:** palliative care research, lifespan, engagement

## Abstract

The ASCENT Consortium was funded by the National Institutes of Health (NIH) in August 2025 with the goal of advancing palliative care (PC) research, evidence, implementation and practice to improve care of persons with serious illness and those who care for them across the lifespan. ASCENT aims to: (1) Develop and coordinate the national scientific infrastructure and community needed to advance PC research, marshalling research expertise currently distributed across research centers and leveraging the impact of that expertise via partnership and collaboration. Partners include persons who have lived experiences with serious illness personally or as caregivers, practicing clinicians, patient advocacy organizations, professional organizations, community organizations, health care systems/settings/payers across the continuum of care and other NIH-funded consortia and networks. (2) Generate new PC research knowledge and methodologies, directly by conducting projects to establish new knowledge or methods that support the work of PC scientists. (3) Foster career development and impact of the PC scientist workforce by funding career development and pilot and exploratory awards, providing access to methodologic consultations and resources such as PC research methodology and career development curricula and facilitating mentoring. (4) Disseminate PC research findings and facilitate subsequent implementation via a multi-pronged approach, including providing resource libraries, guidance documents, best practices, training, and toolkits to facilitate collaboration and co-design with health system partners and relevant organizations. This article describes the goals, organization, resources, programs and activities of the ASCENT Consortium, intending to raise awareness about ASCENT and encourage engagement with, utilization of and collaboration with ASCENT.

## Background

In August 2025, multiple institutes and centers of the National Institutes of Health (NIH) collaborated to fund the Advancing the Science of Palliative Care Across the Lifespan (ASCENT) Consortium. The ASCENT Consortium, funded as a cooperative agreement award (U54) with an initial 5 years of support, is dedicated to advancing palliative care research across the lifespan. It represents the first multi-institute NIH investment and the largest single investment in palliative care research in NIH history.

The establishment of the ASCENT Consortium reflects both decades of progress and a future filled with opportunities and challenges. Over the past 25 years, palliative and hospice care (PC) clinical services have expanded dramatically in the United States (US).^1^ During this period, PC research has also grown, enhancing the scientific underpinnings and evidence base to inform care for people living with serious illness and those who care for them.^2^ At the same time, the US has an increasingly older population and a rising prevalence of serious illness; patients with serious illness across the lifespan continue to experience high levels of suffering. Multiple groups have called for continued development and improvement of evidence-based PC to meet the needs of people with serious illness and those who care for them.^3–5^ Rigorous research is required to identify new, more beneficial treatments and to develop and support more efficient and reliable ways to deliver high-quality PC to all who would benefit across the lifespan regardless of where they receive healthcare. The scientific progress and breakthroughs needed require a multidisciplinary scientific workforce that is appropriately trained and supported, and scientific infrastructure to advance, coordinate, and integrate PC science to improve care for persons across the lifespan living with serious illness and those who care for them.

Several Congressional statements have recognized the importance of PC research, emphasizing “the need for NIH to develop and implement a multi-institute strategy to expand and intensify national research programs in palliative care”.^6^ The Health and Human Services Fiscal Year 2024 Appropriations Bill^7^ designated funds for the NIH to implement a trans-Institute, multidisease strategy to focus, expand, and intensify national research programs in palliative care. The National Institute on Aging (NIA) convened a multi-Institute Palliative Care Research Workgroup^8^ to expand and intensify the strategic coordination of palliative care research efforts and identify future research opportunities. The subsequent release of the “Consortium for Palliative Care Research Across the Lifespan” Notice of Funding Opportunity,^9^ led by the NIA in collaboration with the *Eunice Kennedy Shriver* National Institute of Child Health and Human Development, National Institute of Mental Health, National Institute of Neurological Disorders and Stroke, National Institute of Nursing Research, and National Cancer Institute, created a cross-Institute collaborative structure to leverage synergies, coordinate efforts, develop the scientific workforce, and address remaining gaps in the field of PC. In addition to the Institutes that contributed funding to ASCENT, the NIH Palliative Care Workgroup meets monthly and serves as a resource for the consortium. As a cooperative agreement award, the ASCENT Consortium works collaboratively with NIH subject matter experts from the participating institutes to advance its mission and achieve its goals.

The ASCENT Consortium, funded through this opportunity, seeks to build on the past quarter century of progress while addressing the challenges of the future. In this article, we describe the ASCENT Consortium goals, organization and resources. The intent is to introduce ASCENT and explain how interested individuals and organizations can engage with, utilize, collaborate with and contribute to ASCENT.

### ASCENT Goal, Aims, and Rationale

**Fig. 1.**
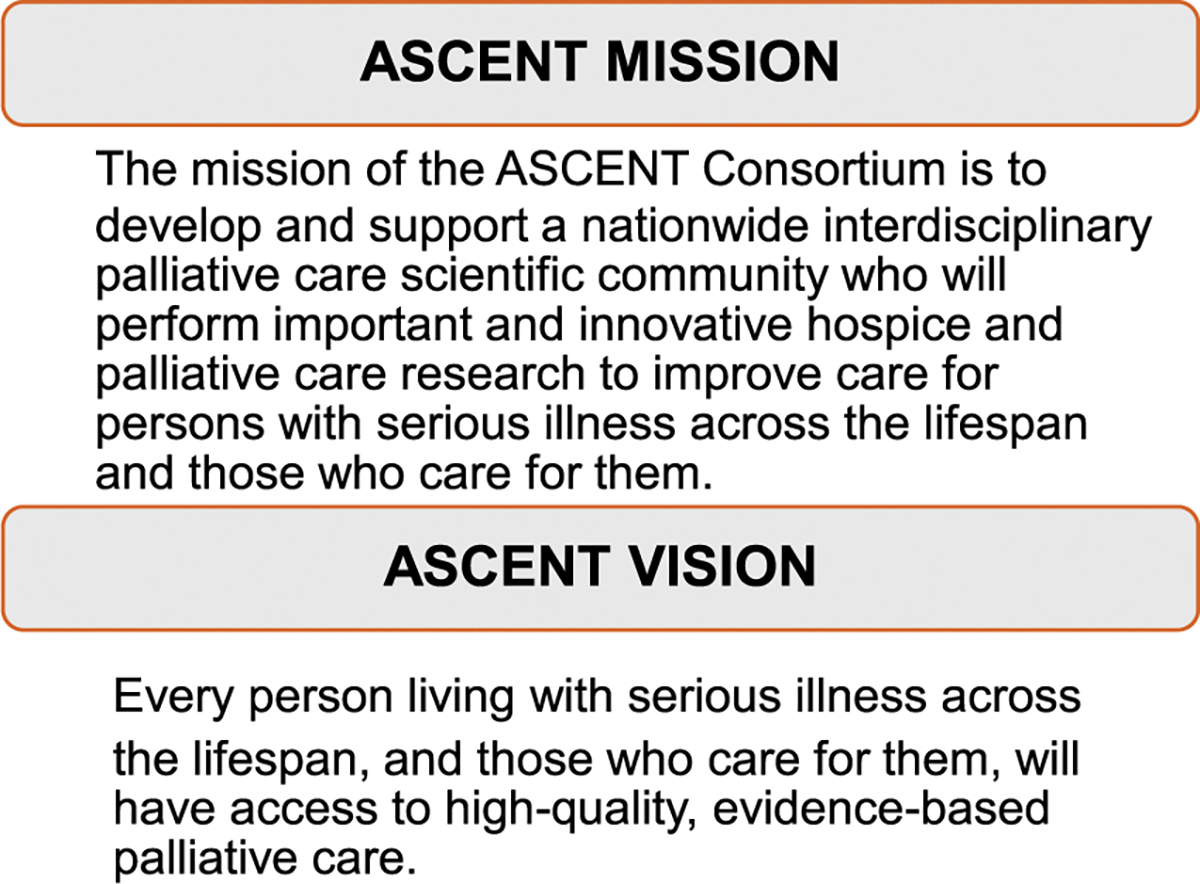
ASCENT mission and vision.

The ASCENT Consortium’s mission and vision (Figure 1) support an overarching goal to **advance palliative care (PC) knowledge, research, implementation and practice.** To achieve this goal, ASCENT is pursuing **four aims:**

1. **Develop and coordinate national scientific infrastructure and community to advance PC research.** ASCENT will marshal research expertise and leverage the impact of that expertise via partnership and collaboration. Partners will include persons who have lived experiences with serious illness personally or as caregivers, practicing clinicians, patient advocacy organizations, professional organizations, community organizations, payers and health care systems and settings across the care continuum and other NIH-funded consortia and networks. Partners will be engaged to inform research priorities and dissemination and translation strategies to maximize the relevance and impact of ASCENT.
2. **Generate new PC research knowledge and research methodologies.** ASCENT will conduct projects to establish new knowledge or research that support the work of PC scientists conducting research across disciplines, diagnoses, populations, settings of care, and lifespan.
3. **Foster career development and impact of the multi-disciplinary PC scientist workforce.** ASCENT will foster career development by funding research scholar and pilot and exploratory awards, providing methodologic consultations, and creating and disseminating PC research methodology and career development curricula and other resources.
4. **Disseminate PC research findings and facilitate subsequent implementation.** To shorten the known 10-to-20-year time lag between the advent of new medical knowledge and implementation into clinical practice, ASCENT will employ a multi-pronged approach, including providing resource libraries, guidance documents, best practices, training, and toolkits, and collaborating with health system partners and relevant organizations to co-design and facilitate subsequent implementation.

### ASCENT ORGANIZATIONAL OVERVIEW

**Fig. 2.**
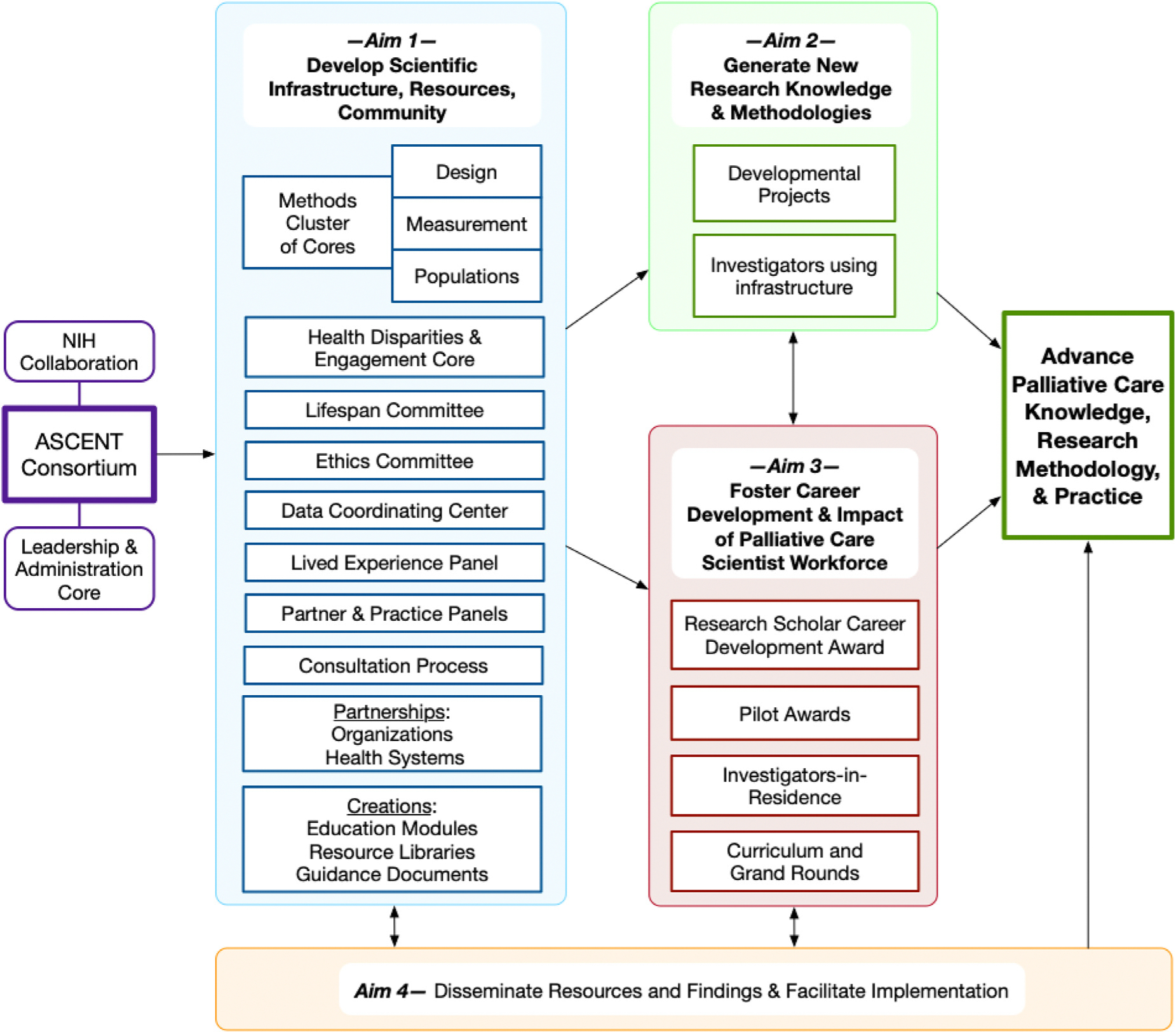
How ASCENT consortium generates new knowledge and advances palliative care research and practice. ASCENT comprises seven cores: Leadership and Administration Core (LAC); Research Design and Methodology (Design) Core; Measure and Measurement Development (Measurement) Core; Population-Based Data (Population) Core; Health Disparities Research and Community Engagement (Health Disparities and Engagement) Core; Research Education Core (responsible for the Research Scholar Awards); and Pilot and Exploratory Grants Core (responsible for Pilot Awards).

ASCENT’s organization and offerings, as described below, are designed to achieve the overarching goal, as illustrated in the organizational diagram presented in Fig. 2. ASCENT has seven foundational Cores: **Leadership and Administrative (LAC), Research Education (REC), Pilot/Exploratory Studies (Pilot), Research Design and Methodology (Design), Measures and Measure Development (Measurement), Health Disparities Research and Community Engagement (Engagement), and Population-based Data (Population).** ASCENT is deliberately organized to assure integration and collaboration across the ASCENT components, leveraging the expertise of Core leadership, members and consultants to effectively advance the field through evidence generation and dissemination. ASCENT currently encompasses ~40 key personnel including the multiple principal investigators, Core leads/co-leads and Core members from more than 20 institutions and a broad range of disciplines. ASCENT also has identified more than 25 consultants and more than 25 initial partners including health systems, community organizations, policy and advocacy groups, clinicians, payers and other NIH-funded research consortia. Each Core will develop and make widely available relevant resources including libraries, guidance documents and tools to support the performance of high-quality PC research and development of the PC scientific workforce.

In addition to the Cores, ASCENT includes several committees and panels to ensure a rich depth and breadth of engagement and focus in key areas. The **Lifespan Committee** will facilitate collaboration and shared learnings across the lifespan, coordinate lifespan-relevant activities, and assure prioritization of lifespan-related research topics. The **Lived Experience Action Panel (LEAP)** includes persons with serious illness and persons with experience caring for those living with serious illness. This panel will provide guidance on priority research areas, participate in review of applications for ASCENT research and training opportunities, and serve as research partners/advisors. The **Practice and Provider Network (PPN)** will seek to leverage clinical practice and health care delivery settings and organizations to serve as locations for the conduct of PC research, disseminate research and training opportunities, nurture collaboration, inform potential research implementation strategies, and provide input on priority research areas.

### ASCENT RESEARCH OPPORTUNITIES

At least half of the ASCENT Consortium’s budget is dedicated to ASCENT’s two annual training and research opportunities: Research Scholar Awards and Pilot and Exploratory Grant Awards. Proposals for these opportunities may employ a range of research methodologies including but not limited to: clinical trials (as defined by the NIH), observational studies, secondary data analyses, health services research, qualitative research, and mixed methods research. Priority areas for these ASCENT research and training opportunities will be determined on an annual basis with input from the LEAP, PPN, and NIH Program Officials and Project Scientists.

**Research Scholar Awards** provide early-career investigators with protected time and training to develop and conduct the research necessary to be competitive for larger, extramurally funded awards. The ASCENT Research Scholar Award funds 2 years of support for salary, research activities, and attending the annual ASCENT scientific meeting, to total no more than $150,000 per year (direct costs), plus allowable facilities and administrative costs. Early-career investigators who seek to establish themselves as palliative care scientists in priority areas that reflect ASCENT goals are encouraged to apply.

**Pilot and Exploratory Awards** provide funding, guidance, and support for studies that provide a foundation for larger, more comprehensive, externally funded research projects, similar to an NIH R21. The ASCENT Pilot and Exploratory Studies Award Program funds up to $250,000 in direct costs per year per award, for a maximum duration of 2 years, plus allowable facilities and administrative costs. The budget may include support for salary, research activities, and funds for attending the annual ASCENT scientific meeting. Although open to investigators at all career stages, promising early-career investigators seeking their first large independent research grant (e.g., K to R transition) and midcareer investigators seeking to obtain a second large research grant to sustain their careers, or explore new research avenues or who are new to palliative care research are encouraged to apply.

### ASCENT CONSULTATION PROGRAM (“ASCENT Lab”)

The ASCENT Consultation Program (the “ASCENT Lab”) aims to support investigators in conducting rigorous and innovative research and to broaden the range of investigators, institutions, geographic areas, populations and disciplines involved in conducting rigorous PC research. The ASCENT Lab facilitates access to ASCENT resources and provides comprehensive and unified guidance to PC investigators. ASCENT Core leads and members and additional experts provide consultations in a variety of formats based on the needs of the individual investigator. The ASCENT Lab program is available to applicants for ASCENT Research Scholar and Pilot and Exploratory opportunities as well as more broadly to the PC research community.

Requests for access to the ASCENT Lab program are placed via the ASCENT website (https://ascentpalliativecare.org/ascent-lab/). When a multi-Core consult is requested, the ASCENT consult team will ensure coordination of recommendations across consultants to avoid conflicting advice and duplication of existing resources and to maximally leverage ASCENT expertise. The ASCENT consult team also will connect investigators with relevant non-ASCENT resources such as the NIA Roybal Centers, IMPACT Collaboratory, NEXT STEPs Network, NIH Pragmatic Trials Collaboratory, Pediatric Palliative Care Research Network, Clin-STAR, the NIH HEAL initiative, NIH workshops and others.

#### ASCENT Grant Review Program:

The grant review program will provide PC investigators who are submitting an NIH K or R01 or equivalent with an anonymous external review and feedback following the applicable NIH review template. The goal is to improve the quality of the science and increase competitiveness for funding. Applicants will submit a request through the ASCENT website; grant review turn-around time will be within approximately 2 weeks from application submission.

### ASCENT RESEARCH TRAINING, EDUCATIONAL, AND METHODOLOGICAL RESOURCES

Multiple **resources and products** will be created and available broadly via the ASCENT website (https://ascentpalliativecare.org/) in a searchable knowledge repository.

Each of the ASCENT Cores has defined deliverables. Examples include:

- Training
  - Online training modules covering key topics in the conception, design, conduct, analysis, and dissemination of PC research across the lifespan, as well as essential career development topics.
  - Virtual PC Research Grand Rounds
- Resources and Guidance
  - Resource Libraries relevant to PC research
  - Guidance documents regarding how to conduct PC research.
  - ASCENT Standard Data Elements
  - PC Measurement Library
  - Guidance for using Medicare and Medicaid data in PC research
  - Best practices for engaging with lived experience panels
  - Best practices for conducting health disparities and community-engaged research
- De-identified data repository
  - Curated repository of data from completed palliative care studies, available for secondary data analyses.

ASCENT also supports an **Investigator in Residence Program**, which provides career development and networking opportunities for early-stage investigators, particularly those from regions and institutions with few PC investigators. Goals of this program are to increase the number of PC investigators, expand the range and capacity of institutions where PC research is conducted, and develop scientific and leadership capabilities leading to future successful grant proposals. Two Investigators in Residence will be assigned to each ASCENT Core annually, selected through a competitive open call for applications. The Investigators in Residence will engage in all Core activities, including knowledge generation, developmental projects, and Core meetings, and will be mentored by a Core member. They will receive guidance in refining a current or future research project and funding to attend the annual ASCENT scientific and business.

### ASCENT GENERATION OF NEW KNOWLEDGE AND RESEARCH METHODS

ASCENT will generate new knowledge and methods through multiple avenues (Figure 2). The evidence emanating from Research Scholar and Pilot and Exploratory Awards will be key to this outcome. It is one of the many reasons why the consortium places a high priority on ensuring the research supported will be highly rigorous, innovative, and advances both the development of the scientific workforce and the corpus of PC science writ large through both investigator mentoring and methodologic support.

A second key knowledge generating aspect is **Developmental Projects.** Developmental Projects, which are intramural to the ASCENT Consortium, have the goal of addressing key research and methodologic challenges in conducting rigorous, impactful PC research. The first cross-consortium developmental project, led by the Design Core in collaboration with ASCENT health care system and organization partners will advance the methodology of conducting PC implementation studies and pragmatic clinical trials in health systems. This project will create an expert panel and a consensus-driven process to generate a guidance document and expert recommendations on strategies for investigators, healthcare systems, and clinical care settings to overcome challenges in conducting high-quality implementation and pragmatic PC studies and inform ASCENT in its grantmaking activities. *Potential* future year (years 2 – 5) Developmental Projects may include: Intervention adaptation for populations at highest risk of poor outcomes and across the lifespan; mixed method data integration; biostatistical challenges (e.g., handling missing data, managing attrition due to death); dyadic and triadic communication study design and analysis; methods to reduce missingness in outcome ascertainment for populations at highest risk of poor health outcomes; and generating standardized estimates of cost and staff time associated with PC service provision using secondary data. All knowledge generation activities will also serve to inform ASCENT consultations and recommendations made therein to investigators.

### ASCENT OUTCOMES

Key outcomes for the ASCENT Consortium include development of research scientists, research productivity, community-building efforts, community resource engagement and production of actionable evidence for dissemination to and implementation by clinicians, policy makers, payers and health systems. Example outcome measures within these categories include: the number and breadth of people and institutions who access ASCENT resources, receive ASCENT funding, or go on to receive independent research funding, publications (including use of new or refined research methods), novel research, and implementation partnerships and networks. Ultimate evidence of success and impact will be uptake of knowledge generated via ASCENT and application to clinical care delivery and policy, thus improving the lives of persons experiencing serious illness and their caregivers across the lifespan.

### HOW TO ACCESS ASCENT RESOURCES

The ASCENT website (https://ascentpalliativecare.org/) is the primary portal of entry and source of information about ASCENT and its resources. We encourage interested individuals to regularly check the ASCENT website for updates. Table 1 provides some examples of potential ways to engage with ASCENT resources.

## Conclusion

ASCENT is an inaugural multi-NIH partnership for advancing PC research across the lifespan that is launching at a pivotal time. For the first time, a Consortium represents ongoing input and coordination from across multiple NIH institutes and centers. Although the needs of people living with serious illness across the lifespan and their caregivers have unique features, ASCENT will capitalize on significant opportunities for shared learning. ASCENT will promote the advancement of PC research across the lifespan and optimize what is learned through an integrative comparative approach. ASCENT will systematically integrate principles of community engagement and rigorous research methods across all Cores and ASCENT initiatives while also providing resources to support, inform, and generate new knowledge related to addressing the needs of populations at highest risk for poor outcomes in the setting of serious illness. ASCENT will have an intentional focus on supporting and developing scientists from multiple disciplines and institutions. ASCENT will help develop the next generation of palliative care scientists and the evidence base for high-quality palliative care practice and policy nationwide.

## Figures and Tables

**Table 1:** *Examples* of How Different Audiences May Interface With ASCENT.

I’m a.....	I’m interested in....	ASCENT Resources and Opportunities

Clinician	Being a study site Serving on ASCENT grant review committees Applying the latest PC evidence to clinical practice	Partner with investigators applying for Research Scholar or Pilot and Exploratory awards Participate in Practice and Provider Network Online educational modules and Grand Rounds Annual Scientific meeting
Early career investigator	Feedback on research ideas and draft grant applications Learning new research methodologies Connecting with other PC investigators Conducting secondary data analyses of available data	Consultation Program (ASCENT Lab) Investigator in Residence Research Scholar Awards Online educational modules and Grand Rounds De-identified Data Repository Population-based Data Core Grant Review Program Annual Scientific meeting Online resources (e.g. Measurement Library, guidance documents)
Senior/experienced investigator	Serving as a consultant or mentor Connecting with other PC investigators Conducting secondary data analyses of available data Getting feedback on my draft grant applications Meeting NIH data sharing requirements for data from closed studies	Consultation Program (ASCENT Lab) Pilot and Exploratory Awards Online educational modules and Grand Rounds De-identified Data Repository Population-based Data Core Annual Scientific Meeting Grant Review Program Online resources (e.g., Measurement Library, guidance documents)
Community or advocacy organization; health care delivery organization; payer; policy maker	Bringing my organization’s perspective to palliative care research Using findings from ASCENT-supported studies to inform clinical care, advocacy, benefit design or policy efforts	Participate in Practice and Provider Network Pilot and Research Scholar Scientific Review Committees Grand Rounds Annual Scientific meeting

## Key Message

The ASCENT Consortium is a new resource to support palliative care (PC) science across the lifespan. PC investigators and those interested in advancing PC science across the lifespan are encouraged to engage with and access the evolving ASCENT resources, available via the ASCENT website (https://ascentpalliativecare.org/).
